# Fluorescent thermal shift-based method for detection of NF-κB binding to double-stranded DNA

**DOI:** 10.1038/s41598-021-81743-1

**Published:** 2021-01-27

**Authors:** Peter D. Leitner, Ilja Vietor, Lukas A. Huber, Taras Valovka

**Affiliations:** 1 Institute of Cell Biology, Biocenter, Medical University of Innsbruck, Innrain 80-82, 6020 Innsbruck, Austria; 2 Austrian Drug Screening Institute, ADSI, Innsbruck, Austria; 3 Department of Biotechnology and Food Engineering, MCI Technik, Innsbruck, Austria; 4 Department of Pediatrics I, Medical University of Innsbruck, Anichstrasse 35, 6020 Innsbruck, Austria

**Keywords:** Biological techniques, Drug discovery

## Abstract

The nuclear factor kappa B (NF-κB) family of dimeric transcription factors regulates a wide range of genes by binding to their specific DNA regulatory sequences. NF-κB is an important therapeutic target linked to a number of cancers as well as autoimmune and inflammatory diseases. Therefore, effective high-throughput methods for the detection of NF-κB DNA binding are essential for studying its transcriptional activity and for inhibitory drug screening. We describe here a novel fluorescence-based assay for quantitative detection of κB consensus double-stranded (ds) DNA binding by measuring the thermal stability of the NF-κB proteins. Specifically, DNA binding proficient NF-κB probes, consisting of the N-terminal p65/RelA (aa 1–306) and p50 (aa 1–367) regions, were designed using bioinformatic analysis of protein hydrophobicity, folding and sequence similarities. By measuring the SYPRO Orange fluorescence during thermal denaturation of the probes, we detected and quantified a shift in the melting temperatures (ΔTm) of p65/RelA and p50 produced by the dsDNA binding. The increase in Tm was proportional to the concentration of dsDNA with apparent dissociation constants (K_D_) of 2.228 × 10^–6^ M and 0.794 × 10^–6^ M, respectively. The use of withaferin A (WFA), dimethyl fumarate (DMF) and p-xyleneselenocyanate (*p*-XSC*)* verified the suitability of this assay for measuring dose-dependent antagonistic effects on DNA binding. In addition, the assay can be used to analyse the direct binding of inhibitors and their effects on structural stability of the protein probe. This may facilitate the identification and rational design of new drug candidates interfering with NF-κB functions.

## Introduction

Nuclear factor κB (NF-κB) is a homo- or heterodimeric transcription factor formed by various Rel proteins, such as p65/RelA, p50, p52, c-Rel, and RelB^1^. NF-κB as a dimer directly interacts with the major groove of DNA recognizing κB consensus sequences in the promoter regions of a variety of inflammatory, immune and stress response genes, thereby regulating their transcription. The binding of NF-κB to DNA is a tightly regulated process that is induced by different stimuli, including pathogens, cytokines, tumor promoters, and chemotherapeutic agents^2^. Physiological NF-κB response is normally transient and is characterized by the efficient termination of NF-κB activity by a number of signalling mechanisms^3^; however, in many pathological situations, such as chronic inflammation and some cancers, NF-κB activity becomes excessive and/or persistent, contributing to the development of disease^4,5^. For this reason, the search for chemical compounds capable of inhibiting NF-κB is considered to be a promising approach for anti-inflammatory and anti-cancer drug discovery^6^. To date, most of such compounds were developed to target the upstream signalling components of the NF-κB pathway, e.g. inhibition of the 26S proteasome, repression of inhibitory κB (IκB) protein kinases (IKKs), and blockage of IκB ubiquitination^7,8^. Because of highly pleiotropic effects of NF-κB inhibitors, there is a strong interest in developing new compounds targeting NF-κB directly and thereby more specifically. Interference with the NF-κB-DNA complex formation by targeting NF-κB molecules is one of the most direct strategies for blocking the NF-κB activity. This approach raises a need for effective methods to monitor the sequence-specific binding of NF-κB to DNA, suitable for high-throughput screening of inhibitory agents.

Numerous techniques exist for studying the interactions between NF-κB dimers and dsDNA. Among them, electrophoretic mobility shift assay (EMSA), DNA foot printing, filter binding and affinity chromatography-based methods are commonly used for qualitative detection of NF-κB binding to the DNA. These conventional and non-equilibrium techniques have limits in their application for the quantitative analysis required for the determination of binding stoichiometries, affinities and kinetics. They also have a number of other disadvantages in terms of timing, sensitivity, reproducibility, and suitability for automatic and high-throughput applications. Recently, several fluorescence-based approaches for measuring DNA-protein interactions have been developed, including “molecular beacon”-based assays, fluorescence polarization assays, protein-DNA FRET assays, and DNA-binding detection based on a modified enzyme-linked immunosorbent assay (ELISA)^9–13^. These techniques are often homogeneous systems allowing quantitative measurement of binding in solution; they are robust, sensitive and can be used in a high-throughput format. However, one key drawback of aforementioned methods is their specific detection of bound and unbound states of nucleic acid, requiring chemical labelling and/or processing of oligonucleotide probes by an additional enzymatic component of the reaction, e.g. exonuclease III^11^. The use of chemically labelled probes not only raises costs, but often limits the range of nucleic acid structures compatible with the assay. Furthermore, the presence of additional enzymatic components restricts the assay conditions and potentially complicates interpretation of results. In particular, the tested compounds might interfere with assay specific enzymes, leading to false positive screening candidates. In addition, current fluorescence-based techniques detect fluorescent signals produced exclusively by the DNA probe without monitoring the integrity (e.g. dimerization) and conformational changes of DNA-binding proteins in the reaction. This may limit the usage of such techniques in various applications, including screening for inhibitors and their validation.

A wide range of biophysical techniques allows monitoring of changes in biochemical and structural properties of proteins, caused by protein-ligand interactions. For instance, the fluorescence-based thermal shift assay (F-TSA) is routinely used for detection of protein-peptide, protein-ion or protein-drug interactions by determining changes in thermal stability of a given protein upon binding its specific ligand^14^. This is achieved by measuring fluorescence produced by the interaction of a specific protein dye, e.g. SYPRO Orange, with hydrophobic core residues, which become exposed during the temperature-induced protein unfolding. Fluorescence values are then used for the determination of the protein transition melting point or melting temperature (Tm) as a measure of thermal stability. Intriguingly, F-TSA has been previously applied for the detection of DNA-mediated changes in the thermal stability of ExsD from *Pseudomonas aeruginosa* and herpes simplex virus type 1 (HSV-1) single stranded (ss) DNA-binding protein ICP8^15,16^. It has been shown that random oligonucleotide ligands caused dissociation of the ExsD trimer in a sequence-independent manner, which was consistent with the loss of protein stability and reduction of melting transition point. The latest was interpreted as the evidence of a non-specific low affinity ExsD-DNA interaction, albeit not confirmed by EMSA^15^. In contrast, the stability of ICP8 was increased upon cooperative binding of ICP8 monomers to ssDNA oligomers of different length. We made an initial observation that short dsDNA oligomers possessing the κB consensus sequences 5′-GGGRNYYYCC-3′ (in which R is a purine, Y is a pyrimidine, and N is any nucleotide) increased the Tm of p65/RelA-specific Rel homology domain (RHD)^17^. These observations suggested that the measurement of thermal stability could be suitable to set up an assay for detecting highly specific and sequence-dependent interactions of NF-κB with dsDNA.

Herein, we report the development and evaluation of a simple, robust, quantitative, high-throughput suitable method for in vitro studies of the NF-κB DNA-binding activity using the homogenous fluorescence-based thermal shift (F-TSA) assay with standard real-time PCR equipment. We also demonstrate its applicability and high efficiency for the identification of compounds disrupting NF-κB-dsDNA interactions and for the simultaneous monitoring of their direct effects on the structural stability and folding of dimeric NF-κB proteins.

## Results

### Design of NF-κB protein probes and assessment of their thermal stability

**Figure 1:**
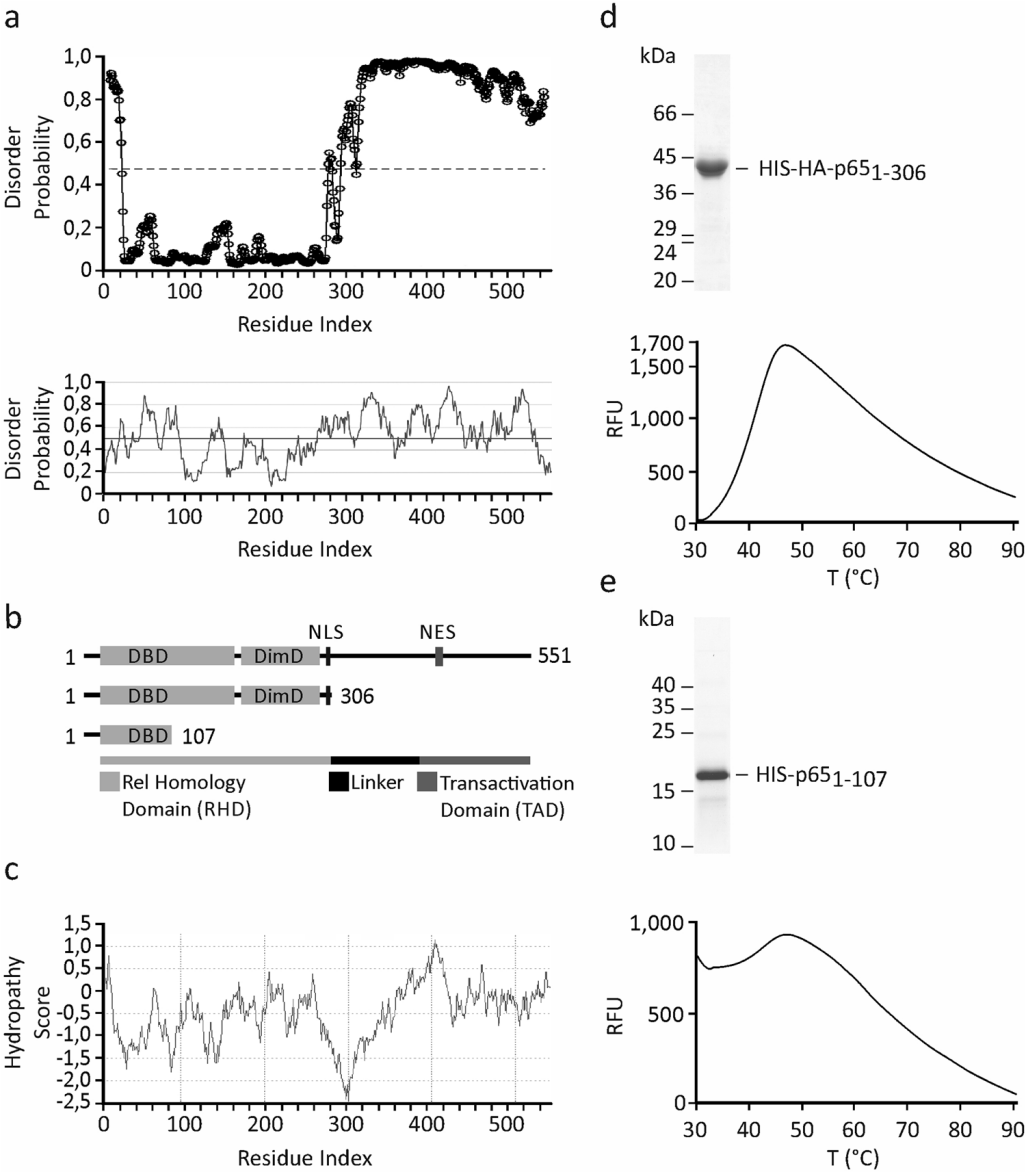
Design, purification and thermal stability of p65/RelA peptide probes. (**a**) Prediction of protein disorder using the SPOT-disorder (upper) and IUPred (lower) web servers for the p65/RelA subunit of NF-κB. The IUPred output was generated using the long disorder option. (**b**) Scheme of the p65/RelA protein and its deletion variants representing the NF-κB probes. The DNA-binding (DBD) and dimerization (DimD) subdomains, and nuclear import (NLS) and export (NES) signals are indicated. (**c**) The hydrophobic properties of p65/RelA were analysed using the ExPASy Protscale web server. (**d**,**e**) SDS-PAGE analysis of HIS-tagged p65_1-306_ and p65_1-107_ proteins expressed in *E. coli* and purified by affinity chromatography. The thermal stabilities of purified proteins were analysed by measuring SYPRO Orange dye fluorescence over a temperature (T) range of 25–95 °C using a real-time PCR thermocycler. Representative unfolding curves are shown. *RFU* relative fluorescence unit.

The thermofluor assay with a SYPRO Orange dye is compatible with most soluble proteins that are well folded and characterized by a relatively large hydrophobic core. Intrinsically disordered proteins generate a high background fluorescence caused by fluorophore binding to the protein in its native state, interfering with the assay. To design protein probes compatible with this method, we analysed the amino acid sequence of the p65/RelA subunit of NF-κB for the presence and distribution of potentially disordered or structurally flexible segments using two different trained algorithms: SPOT-disorder and IUPred2A (http://sparks-lab.org/server/SPOT-disorder/, https://iupred2a.elte.hu)^18,19^. SPOT-disorder and IUPred2A predicted extensive segments of disordered structure (score above 0.5) within the region surrounding the nuclear localization signal (NLS), the linker region and the C-terminal transactivation domain (TAD): amino acids 293–304 and 312–551 (Fig. 1a,b). This correlated well with the structural studies employing CD and NMR spectroscopy that showed a random coil conformation of the C-terminal TAD^20^. X-ray structures from the PDB (1MY7, 2RAM, 5U01) indicated that the RHD of p65/RelA (aa 19–304) consists of two globular regions exhibiting an immunoglobulin (Ig)-like fold. These two folded regions are joined by a short, approximately 10 amino acids in length, flexible sequence and represent DNA-binding and dimerization subdomains^21^. Both algorithms correctly predicted a high degree of folding for the N-terminal sequence comprising residues 15–293, although IUPred2A showed that the sequence within the DNA-binding subdomain (aa 24–92) had some propensity for being unstructured. It is possible that the residues beyond this sequence are important for promoting folding, seen in the crystal structure, likely via dimerization mediated by the remaining part of RHD^22^. Next, we analysed the hydrophobic properties of p65/RelA using Kyte and Doolittle method^23^ on ExPASy Protscale (https://web.expasy.org/protscale/)^24^. The hydropathy plot revealed that the p65/RelA protein was moderately hydrophilic with the grand average of hydropathy (GRAVY) value of − 0.463. We also found that hydrophobic patches were evenly distributed along the protein sequence with the exception of boundary region between the structured N-terminal and intrinsically disordered C-terminal parts. This region surrounded the NLS and was characterized by a low hydropathy score (Fig. 1c).

**Figure 2:**
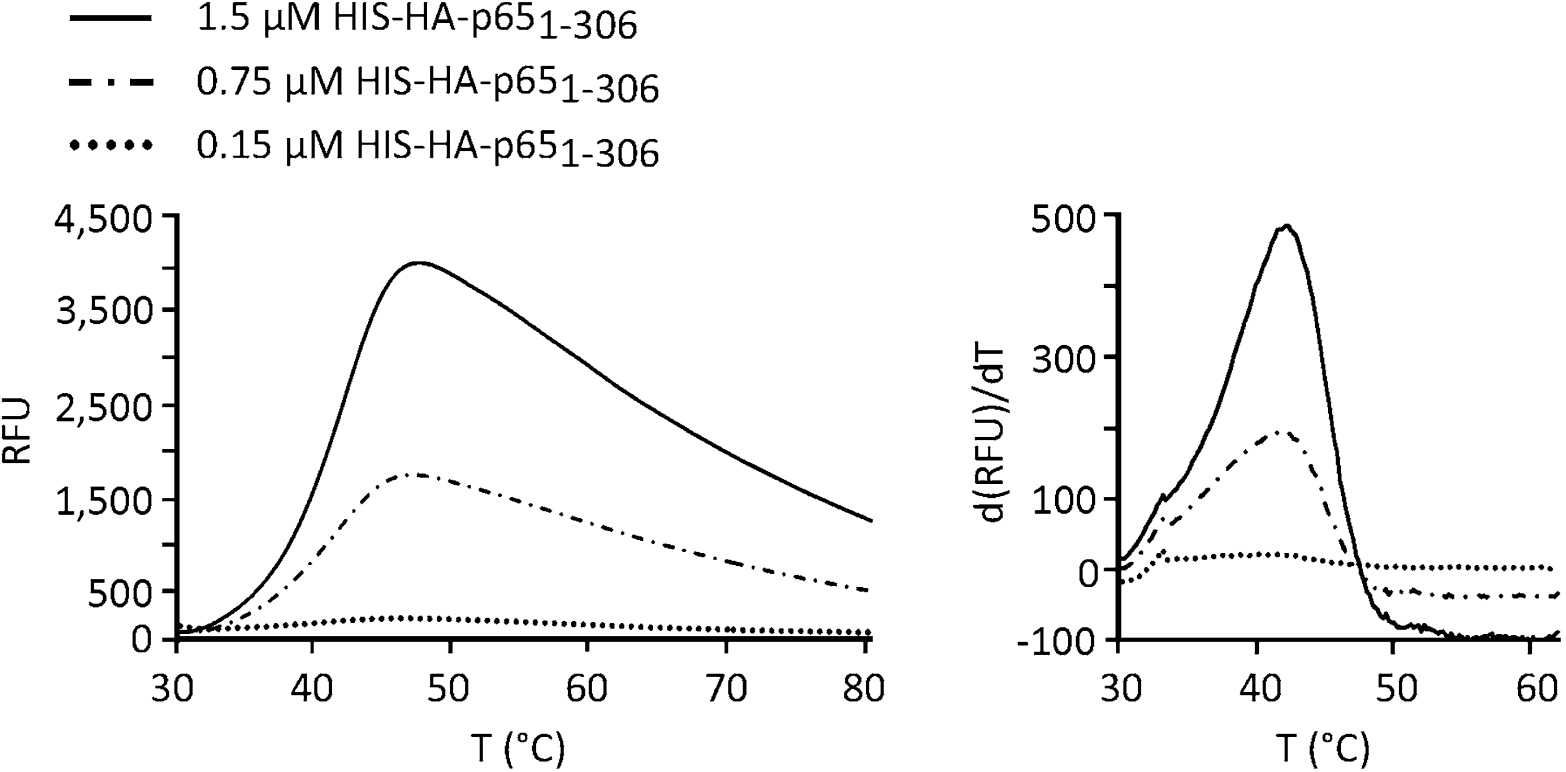
P65/RelA probe detection limit by using SYPRO Orange dye. The thermal denaturation was evaluated for a concentration series of p65_1-306_ peptide using SYPRO Orange dye as in Fig. 1d. Representative unfolding (left) and positive derivative (d(RFU)/dT) (right) curves are shown. *RFU* relative fluorescence unit.

Based on our in-silico analysis and available structural data, we designed two different NF-κB protein probes for the F-TSA-based DNA-binding assay. Both probes were HIS-tagged truncated versions of the human p65/RelA subunit consisting of either the complete RHD (p65_1-306_) or its N-terminal DNA-binding part (p65_1-107_) (Fig. 1b). The p65_1-306_ probe could form DNA-binding competent p65/RelA dimers as shown by EMSA^17^. In contrast, the p65_1-107_ protein was expected to be purified as a monomer due to the lack of dimerization subdomain. Thus, it could be useful for controlling the formation and stability of p65/RelA dimers in the assay. Both proteins were Ni–NTA affinity purified from *E. coli* and their thermal stabilities were initially analysed using the SYPRO Orange fluorescence dye (Fig. 1d,e). Comparable amounts of p65_1-306_ and p65_1-107_ proteins (0.75 µM) were thermally denatured in the presence of SYPRO Orange dye using a real-time PCR thermocycler. Analysis of the unfolding curves showed that p65_1-107_ was characterized by a high initial fluorescence, which exhibited only a minor increase upon heating. High fluorescence background produced by p65_1-107_ protein could possibly be explained by its structural instability or partially unfolded state consistent with the IUPred2A prediction. However, this prevented the further use of p65_1-107_ as a probe for the F-TSA assay. Unlike the N-terminal part, the unfolding curve of p65_1-306_ peptide was characterized by a low initial fluorescence that gradually increased with temperature reaching the maximal value at approx. 42.5 °C. This value was defined as the Tm of p65_1-306_ under specified experimental conditions. We also used serial dilutions of p65_1-306_ to evaluate the optimal amount of p65_1-306_ probe for the reliable detection of protein unfolding and precise estimation of Tm in the F-TSA assay (Fig. 2). We found out that 0.15 µM of p65_1-306_ represented the lowest limit of protein detection but was insufficient for the correct estimation of Tm whereas 0.75 µM and 1.5 µM concentrations produced high-intensity fluorescent signals suitable for this analysis.

RHD proteins share a high degree of similarities in their structural organization and amino acid sequences. Similar to p65, we also generated and purified HIS-tagged truncated version of the human p50 subunit consisting of the complete RHD (aa 1–367). The p50_1-367_ protein was characterized by a higher Tm (approx. 44.5 °C) than for p65 and can be used as an alternative NF-κB probe in TSA-based applications (Supplementary Fig. 1).

### Detection of sequence-specific dsDNA binding by measuring the thermal stability of NF-κB

**Figure 3:**
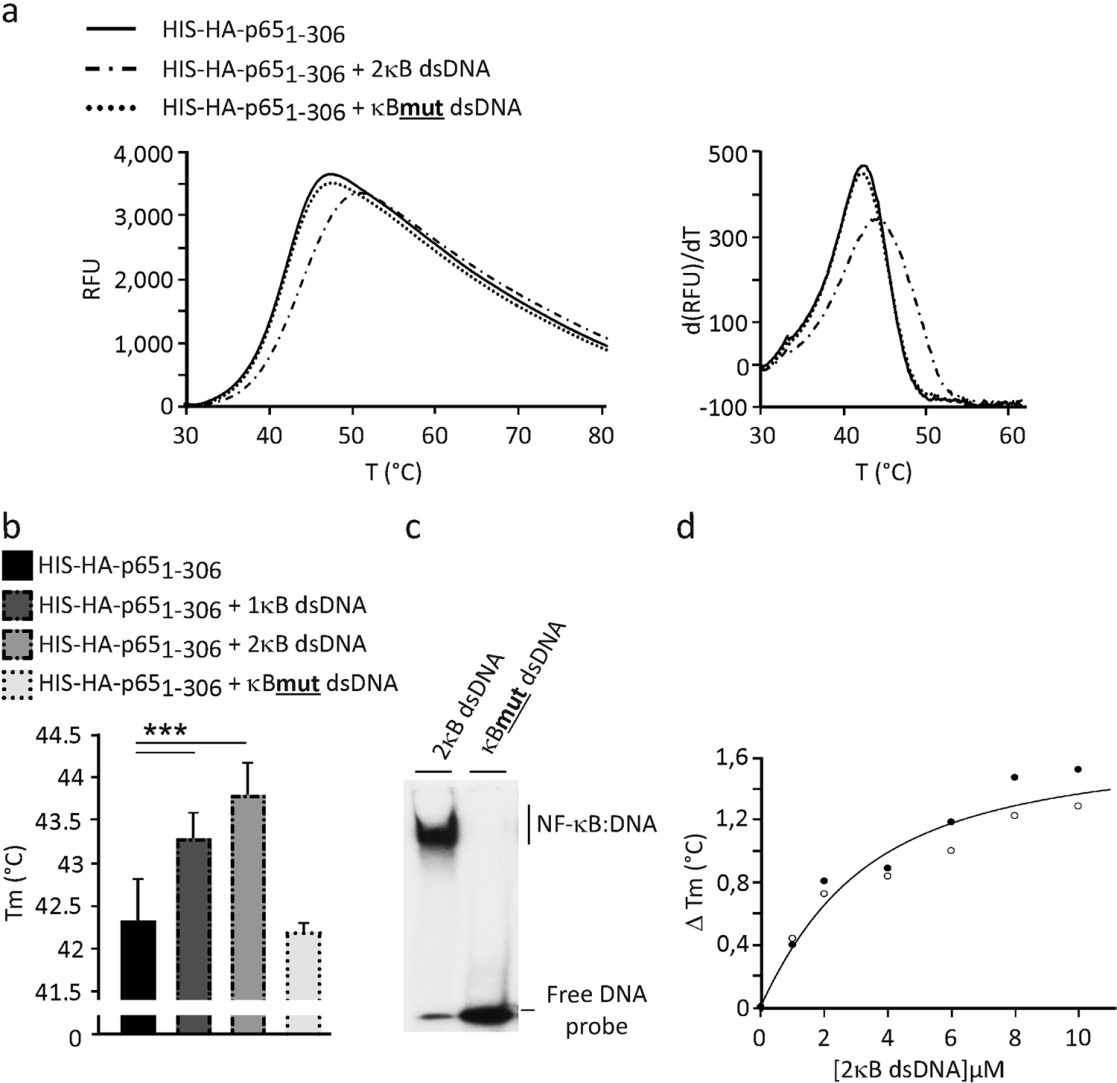
Detection of sequence-specific dsDNA binding to p65/RelA probe in the thermal shift assay. (**a**,**b**) The κB-dependent thermal stabilization of p65_1-306_ probe in the presence of 10 µM dsDNA oligomers. Representative unfolding (left) and positive derivative (d(RFU)/dT) (right) curves are shown. Midpoint temperatures of the protein-unfolding transition (Tm) are presented as bars. Values are mean ± SD of at least three independent measurements (***p < 0.001). (**c**) EMSA of p65_1-306_ protein (0.5 µM) using ^32^P-labelled 2κB and mutated κB consensus oligonucleotides (1 × 10^4^ cpm). (**d**) Thermal stability shift assessed with varying 2κB dsDNA concentrations. ΔTm, change in Tm of p65_1-306_ probe caused by dsDNA. The curve is based on two separate acquisitions shown as open and closed circles.

Next, we tested how the thermal stability profiles of p65_1-306_ and p50_1-367_ proteins were affected by the presence of dsDNA either with one (1κB dsDNA) or two identical κB binding sites (5′-GGGACTTTCC-3′) separated by four base pairs (bp) (2κB dsDNA). When 10 µM of each of the ds oligonucleotides were incubated with 1.5 µM p65_1-306_ or p50_1-367_ probes prior to the thermal denaturation, a shift in the protein melting temperatures was observed that raised the Tm by approx. 1–1.5 °C to 43.5–44 °C or 2–3 °C to 46.5–47.5 °C, respectively (Fig. 3a,b, Supplementary Fig. 1). The increase in thermal stability seen for the NF-κB proteins was explained by the formation of additional bonding contacts between dsDNA helix and the DNA-binding residues of RHD^21^. The presence of the second NF-κB-binding motif in 2κB dsDNA had only a moderate additive effect on the increase in protein Tm when compared to 1κB dsDNA. This likely to be explained by the anti-cooperative binding of RHD dimers previously reported for dsDNA, possessing two tandemly arranged κB sites^25^. To verify that the stabilization effect was solely caused by the sequence-specific binding of NF-κB to dsDNA, we designed an oligonucleotide duplex lacking the cognate binding sites (κBmut dsDNA). The inability of p65_1-306_ dimers to bind the κBmut dsDNA was confirmed by EMSA (Fig. 3c). Consistent with this result, the κBmut dsDNA had no detectable effect on the stability of p65_1-306_ (Fig. 3a,b)_,_ indicating high specificity for the detection of κB consensus-dependent dsDNA binding.

Next, we applied the assay for evaluating the DNA-binding activity of recombinant p65_1-306_ and p50_1-367_ proteins. For this purpose, 1.5 µM of each of the recombinant proteins were incubated with different testing setups. As shown in Fig. 3d and Supplementary Fig. 1, the presence of 2κB dsDNA increased the Tm of p65_1-306_ and p50_1-367_ in a concentration-dependent manner, resulting in an estimated ΔTmax equal to 1.678 °C and 2.462 °C, respectively. Considering that the p65_1-306_- and p50_1-367_-specific dsDNA binding involved two equivalent κB sites, the calculated dissociation constants (K_D_) were 2.228 × 10^–6^ M and 0.794 × 10^–6^ M. This is consistent with previous reports demonstrating higher DNA-binding affinities for p50 homodimers^26,27^. Thus, our analysis demonstrated the feasibility of F-TSA DNA-binding assay for a quantitative evaluation of equilibrium NF-κB-dsDNA interactions.

Because different experimental tasks may require the testing of DNA binding at various temperatures, we compared the stability and dsDNA binding of p65_1-306_ probe at 4 °C and 30 °C. As shown in Supplementary Fig. 2, there was no significant difference in the thermal stability of the probe incubated with or without dsDNA at 4 °C and 30 °C. This permits the testing of DNA binding in a broad range of temperatures, providing a substantial flexibility for experimental setups.

### F-TSA-based analysis of dsDNA binding inhibition by NF-κB-targeting compounds

**Figure 4:**
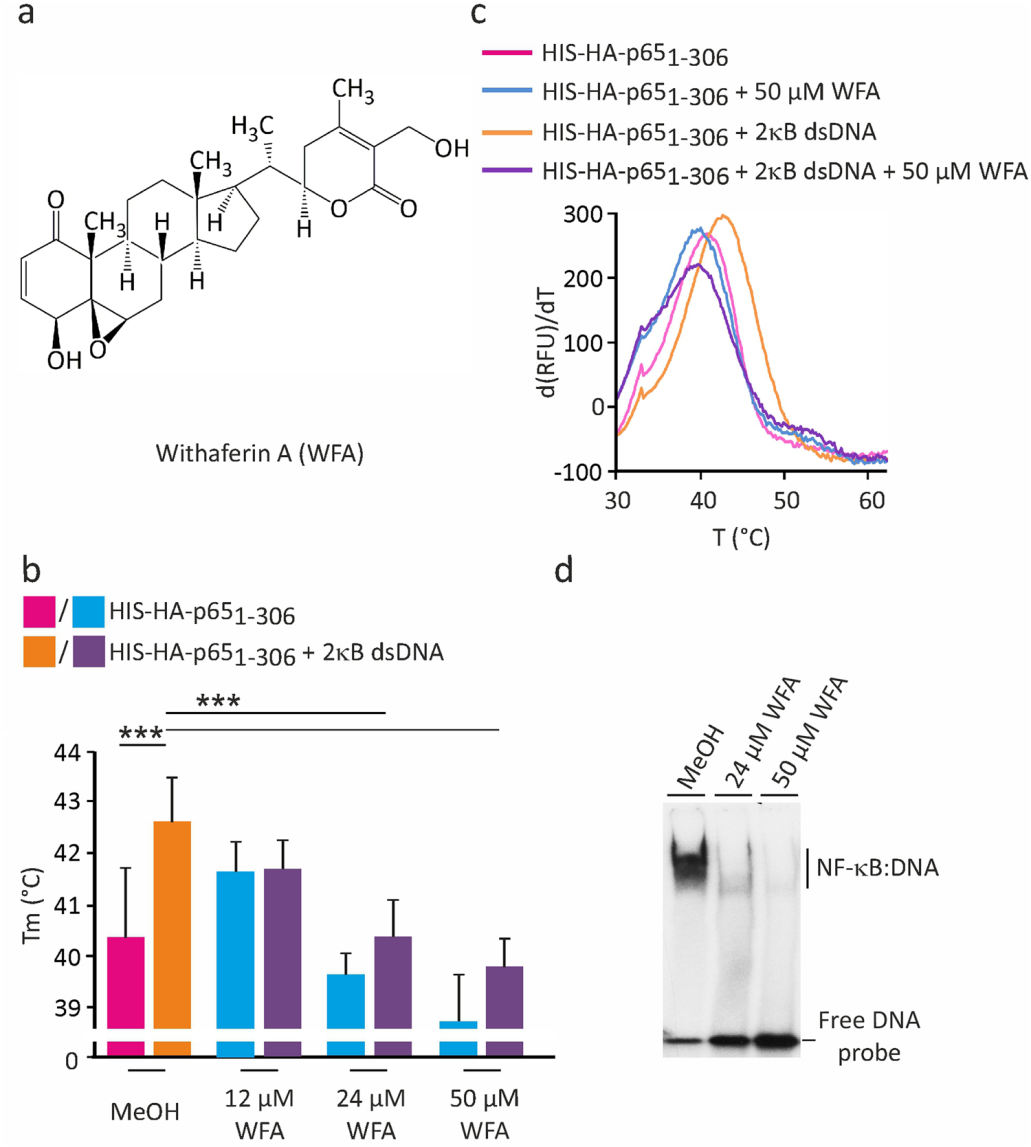
Effect of withaferin A on the dsDNA-induced p65/RelA thermal stability shift. (**a**) Chemical structure of WFA. (**b**,**c**) The thermal denaturation of p65_1-306_ was measured in the absence and presence of 10 µM 2κB dsDNA and varying concentrations of WFA, as indicated. Representative derivative (d(RFU)/dT) curves are shown for untreated and treated with 50 µM WFA samples. Midpoint temperatures of the protein-unfolding transition (Tm) are presented as bars. Values are mean ± SD of at least three separate measurements (***p < 0.001). (**d**) The inhibitory effect of WFA on the DNA-binding activity of p65_1-306_ was verified by EMSA.

**Figure 5:**
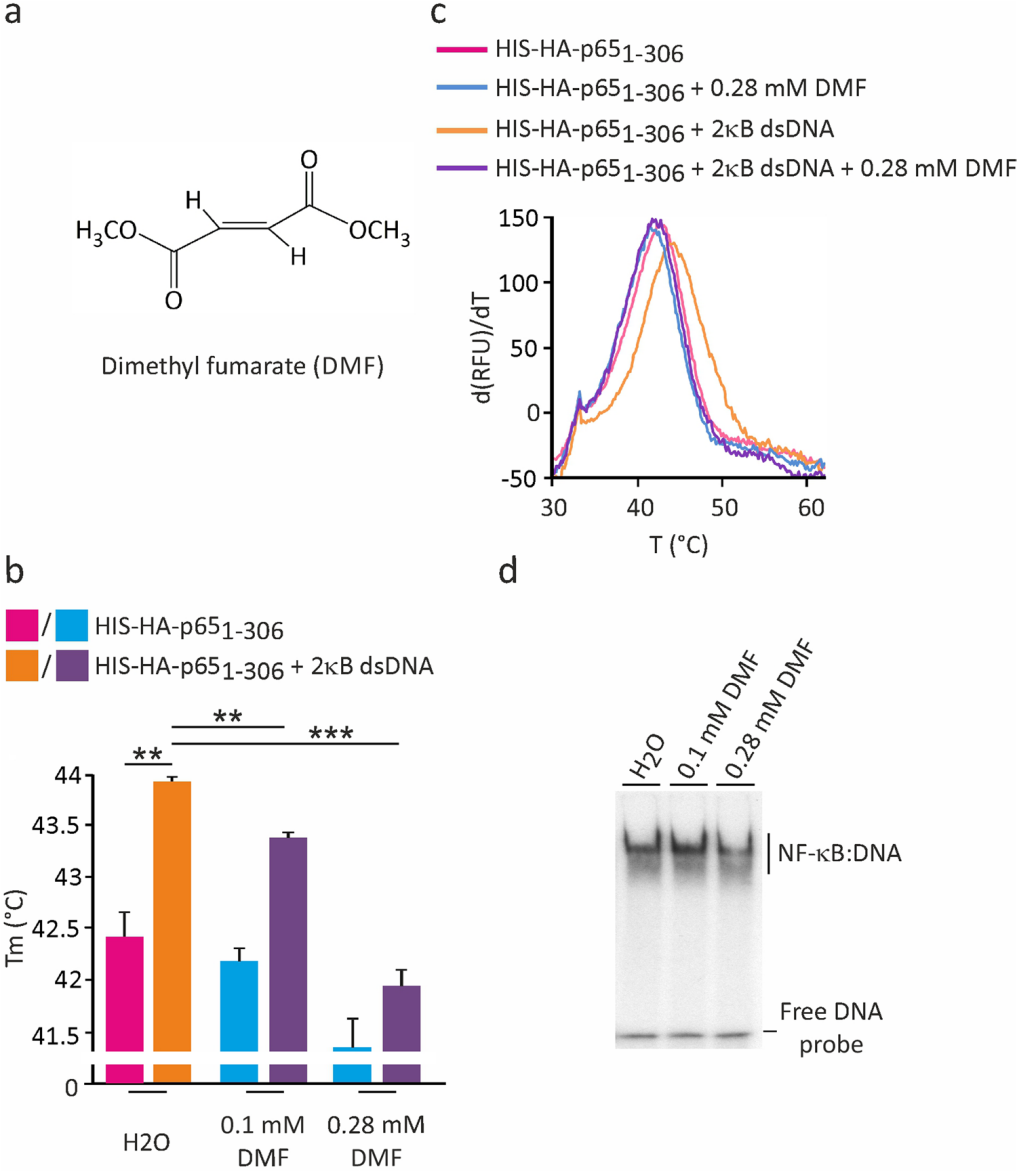
Dose-dependent inhibition of p65/RelA dsDNA binding by dimethyl fumarate. (**a**) Structure of DMF. (**b**,**c**) The thermal denaturation of p65_1-306_ was evaluated as in Fig. 4b with indicated concentrations of DMF. Positive derivative (d(RFU)/dT) curves are shown for control and treated with 0.28 mM DMF samples. Midpoint temperatures of the protein-unfolding transition (Tm) are presented as bars. Values are mean ± SD of three measurements (**p < 0.01 and ***p < 0.001). (**d**) EMSA analysis of p65_1-306_ DNA binding in the presence of DMF.

We used the known NF-κB inhibitors WFA and DMF to further investigate the utility of the F-TSA dsDNA-binding assay for screening and validation applications (Figs. 4, 5). Their chemical structures are shown in Figs. 4a and 5a, respectively. Both compounds interfere with the DNA-binding activity of NF-κB, yet utilizing distinct inhibitory mechanisms^28,29^. DMF covalently modifies reactive Cys38 of p65/RelA that forms a hydrogen bond with the sugar/phosphate DNA backbone, whereas WFA binds to the dimerization interface and surface residues E285 and Q287 of p65/RelA homodimer, inhibiting dimerization and DNA binding directly and allosterically. The thermal stability of p65_1-306_ was evaluated in the presence of different concentrations of inhibitor alone or together with the 2κB dsDNA. We found that both tested compounds reduced the thermal stability of p65_1-306_ dimer in a concentration-dependent manner, i.e. 24 µM and 50 µM WFA decreased the Tm by approx. 0.7 and 1.7 °C (Fig. 4b,c); 0.1 mM and 0.28 mM DMF by approx. 0.3 and 1.1 °C (Fig. 5b,c), respectively. Loss of thermal stability mediated by WFA and DMF indicated their direct binding and/or modification of p65_1-306_ protein associated with destabilizing structural changes. Furthermore, the inhibitory drugs substantially reduced the thermal stabilization effect caused by the 2κB dsDNA, indicating the interference with the DNA-binding activity of p65_1-306_ protein. These effects were dose-dependent and correlated well with the WFA- and DMF-specific inhibition of DNA binding, as confirmed by the EMSA assay (Figs. 4d, 5d). Interestingly, the indicated WFA concentrations also inhibited the DNA-binding activity of p50_1-367_ (Supplementary Fig. 3). We investigated the WFA-mediated inhibition of p65 and p50 proteins in more detail using a serial dilution of the inhibitor. In these experiments we found that WFA inhibited the DNA-binding activities of p65_1-306_ and p50_1-367_ with IC_50_ of approx. 16.4 µM and 19.1 µM, respectively (Supplementary Fig. 3). It is possible, however, that the transactivation (TAD) domain missing in our probes may affect the WFA-mediated inhibition. Thus, we cannot exclude that the actual IC_50_ values differ for the intact p65 and p50 proteins. Additionally, we tested a more general inhibitor of transcription factors the organoselenium compound *p*-XSC^30^ and used the betaine lipid DGTS^31^ as a negative control. The later one acts as an anti-inflammatory agent that inhibits nitric oxide (NO) production in macrophage cells through downregulation of inducible nitric oxide synthase (iNOS). As expected, *p*-XSC inhibited the dsDNA-mediated stabilization of p65_1-306_ in a dose-dependent manner (Supplementary Fig. 4). In contrast, even a high concentration of DGTS showed no detectable effect on the DNA-binding activity of p65, supporting specificity of the assay (Supplementary Fig. 4). Thus, our results highlighted the amenability of the F-TSA-based DNA-binding assay for the screening of small molecules as modulators of NF-κB-specific interactions with the dsDNA.

## Discussion

In this study we describe a fluorescence-based, label-free method for the quantitative detection of NF-κB binding to κB dsDNA using the thermal stability shift assay. This approach allowed the direct evaluation of NF-κB-dsDNA interaction by monitoring an increase in the thermal stability of protein upon sequence-specific DNA binding. Firstly, we present here a concept for the design and optimization of the recombinant NF-κB protein probes using a selection of freely available web servers for the sequence-based structural analysis. As shown here for the p65_1-306_ and p65_1-107_ variants of p65/RelA protein, the evaluation of several structural parameters, including disorder probability and hydropathy index, allowed us to predict the probe suitability for the assay. Similar algorithm is likely to be useful in designing peptide probes for other DNA-binding proteins.

Secondly, we optimized the assay and verified its accuracy and sensitivity in various experimental setups. The established assay takes advantage of the high-intensity fluorescence produced upon binding of SYPRO Orange dye to exposed hydrophobic amino acid residues, enabling the detection of protein in the nanomolar range. These protein concentrations are comparable to those used in EMSA^25^ and various different fluorescence-based methods^11,32^. The sensitivity of this assay might be further improved by using different fluorescent dyes with a different mode of binding to the protein probe. One such alternative could be the use of thiol-specific fluorochrome N-[4-(7-diethylamino-4-methyl-3-coumarinyl)phenyl]maleimide (CPM), that modifies cysteines embedded in the protein interior, thereby triggering the emission of light^33^. P65_1-306_ contained nine cysteine residues as potential modification sites for CPM, supporting the use of this “off-on” fluorescent compound in the proposed assay. Moreover, we showed that the thermal stabilization of p65_1-306_ probe by the presence of dsDNA (ΔTm ~ 1.5 °C) was highly specific for the κB consensus sequence with virtually no binding to mutated κB oligos. The titration of dsDNA concentrations permitted an accurate calculation of the dissociation constants for p65 as well as for a homologous protein p50, K_D_ = 2.228 × 10^–6^ M and 0.794 × 10^–6^ M. The values were consistent with distinctive DNA-binding affinities previously observed for these proteins in vitro^26,27^. These results proved the suitability of the NF-κB DNA-binding assay for the equilibrium analysis and determination of basic dsDNA-binding parameters. However, one has to consider that the binding constants obtained by the F-TSA-based assay rely on the detection of DNA binding at temperatures higher than physiological ones.

Finally, we verified the suitability of this method for screening and characterization of small molecules interfering with the NF-κB binding to DNA. The novel assay presented here measured the gradual inhibition of p65-specific Tm stabilization by dsDNA in the presence of increasing amounts of NF-κB-targeting compounds, i.e. withaferin A, dimethyl fumarate and p-xyleneselenocyanate. We also discovered here that WFA inhibited the DNA-binding activity of p50, suggesting its general inhibitory effect on Rel homology domain proteins. Interestingly, the WFA- and DMF-mediated inhibition correlated to a large extent with the loss of p65/RelA and p50 stabilities, suggesting structural changes introduced by these inhibitors. The structural re-arrangements of RHD upon WFA binding to the hydrophobic core domain (HCD) of p65/RelA have been previously suggested as possible explanation for its allosteric inhibitory effect, albeit missing direct experimental evidence. Our findings provide first experimental support of this hypothesis. DMF acts differently to WFA and is known to covalently modify Cys38 of p65/RelA interfering thereby with the hydrogen bond formed between this residue and the sugar/phosphate DNA backbone. The data obtained in our assay suggested that along with the H-bond formation interference, the structural changes in p65/RelA might be relevant to the DMF-specific inhibitory mechanism. Hence, our approach allows a direct and simultaneous evaluation of interactions between NF-κB and dsDNA as well as NF-κB and inhibitory compounds, providing additional insights on possible interference mechanisms. We envision that our newly developed assay could be expanded to detect and characterize sequence-dependent protein-dsDNA interactions mediated also by other transcriptional factors.

## Methods

### Recombinant protein expression and purification

Bacterial pET28b(+) and pET21b(+) plasmids coding for HIS-tagged p65_1-306_ and p65_1-107_ were described previously^17^. The coding region of p50 (aa 1–367) was cloned into the pcDNA3.1(+)-MYC vector to produce the N-terminal MYC-tagged p50 version. The MYC-tagged p50 fragment was fused to HIS-tag coding sequences by subcloning into the BamHI and XhoI sites of the pET28b(+) vector (Novagen). Recombinant proteins were expressed in *Escherichia coli (E. coli)* strain BL21 (DE3) CodonPlus-RIL (Stratagene) and purified by affinity chromatography on an ÄKTA Purifier System using HisTrap column (GE Healthcare). In brief, transformed bacteria were incubated in LB media at 37 °C up to the OD_600_ = 0.8 and then induced by 0.3 mM IPTG for 72 h at 4 °C. Bacteria were centrifuged at 9,000×*g* for 20 min at 4 °C. The bacterial pellets were resuspended in ice-cold *E. coli* LB buffer (50 mM Tris–HCl pH 7.5, 500 mM NaCl, 5% glycerol, 0.5% NP-40, 40 mM imidazole, 1 mM PMSF, 2 mM β-mercaptoethanol) and lysed using the French Press (HTU-DIGI-F-Press F-013, Heinemann) at 1,300 psi. To reduce viscosity, cell extracts were supplemented with 1.7 μg∕ml DNase I and 0.3 mM MgCl_2_ and incubated for 35 min on ice. The samples were then clarified by centrifugation at 18,000×*g* for 20 min at 4 °C and subsequently loaded onto a 1 ml HisTrap column equilibrated in *E. coli* LB2 buffer. Column was washed thoroughly with washing buffer (50 mM Tris–HCl pH 7.5, 500 mM NaCl, 5% glycerol, 0.5% NP-40, 60 mM imidazole, 2 mM β-mercaptoethanol) followed by washing with the same buffer without NP-40. Bound proteins were eluted with elution buffer (50 mM Tris–HCl pH 7.5, 500 mM NaCl, 5% glycerol, 500 mM imidazole, 2 mM β-mercaptoethanol) at a flow rate of 0.5 ml/min and dialyzed overnight against the same buffer without imidazole. Protein purity was checked by 10% and 15% SDS-PAGE and concentrations were determined by using the Coomassie Plus Protein Assay Reagent (Thermo Scientific). Protein aliquots were stored in liquid nitrogen.

### Electrophoretic mobility shift assay

Complementary oligonucleotides were synthesized and HPLC-purified by Sigma-Aldrich. Oligomer duplexes were prepared by mixing 100 pmol of each of the two complementary oligonucleotides in a total volume of 50 µl of annealing buffer (10 mM Tris HCl pH 8.0, 5 mM MgCl_2_, 65 mM KCl 0.5 mM EDTA, 1 mM DTT). After 3 min incubation at 95 °C, the samples were left to form oligomer duplexes while gradually cooling to room temperature. dsDNA oligomers were 5′-^32^P-labeled with T4 DNA polynucleotide kinase (New England Biolabs) and [γ-^32^P]ATP (Perkin Elmer, 3,000 Ci/mmol) followed by removal of the excess of radioactive ATP using LiCl/ethanol precipitation. Recombinant p65_1-306_ protein (0.5 µM) was incubated in EMSA buffer containing 10 mM Tris HCl pH 7.5, 0.5 mM EDTA, 65 mM KCl, 5 mM MgCl_2_, 1 mM DTT, 100 μg/mL BSA, 10% glycerol, 50 ng/µl poly(dI-dC), and ^32^P-labeled ds oligonucleotide (2κB dsDNA: 5′-AATTCGGGACTTTCCCGTCGGGACTTTCC-3′; κBmut dsDNA: 5′-AGCTCGCTATTAGACAGCTGCTATTAGACTGCA-3′) for 45 min at 30 °C. Protein-DNA complexes were resolved by native 4% PAGE and visualized by autoradiography.

### Fluorescence-based thermal shift assay

The thermal shift assays were performed using the PikoReal 96 Real-Time PCR System (Thermo Scientific) melting curve program with a temperature increment of 0.2 °C and a temperature range of 25–95 °C. All reactions were incubated in a 25 μl final volume and assayed in 96-well plates using 1:1,000 dilution of 5,000 × SYPRO Orange stock solution (Sigma-Aldrich) and indicated concentrations (0.15–1.5 µM) of recombinant protein probes diluted in buffer containing 50 mM Tris·HCl pH 7.5, 225 mM NaCl, and 2.25% glycerol. The 1κB (AGTTGAGGGGACTTTCCCAGGC), 2κB and κBmut dsDNA oligomers (1–10 µM) were added to reaction to assess the DNA binding-dependent thermal stabilization of proteins. Oligomers and inhibitors (WFA dissolved in methanol; DMF dissolved in H_2_O (Sigma-Aldrich)) were incubated with proteins at 4 °C for 25 min prior to acquiring the melting curves. 1,4-phenylene-bis(methylene)selenocyanate (*p*-XSC) (Abcam) and diacylglyceryl- N, N, N-trimethylhomoserine (DGTS) (Sigma-Aldrich) were dissolved in DMSO and incubated with the p65_1-306_ probe at ambient temperatures for 30 min prior to the analysis. Positive derivative (d(RFU)/dT) curves were built and the midpoint temperatures of the protein-unfolding transition (Tm) were estimated using the PikoReal 96 Real-Time PCR System software (Thermo Scientific).

### Data analysis

Results are presented as mean ± SD for variations between separate measurements. Statistical comparisons were calculated using the Student *t* test. *P* values are denoted as follows: *P < 0.05, **P < 0.01, and ***P < 0.001. K_D_ determination was obtained by plotting the change in Tm (ΔTm) as a function of dsDNA concentrations ([dsDNA] or [D]). To fit the parameters, the curve fit function from the Python 3.5 library Scipy Optimize was applied. Equation ${\Delta }Tm = \frac{{\left( {{\Delta }T_{max} + \left[ {\text{D}} \right] + {\text{K}}_{D} } \right) - \sqrt {\left( {{\Delta }T_{max} + \left[ {\text{D}} \right] + {\text{K}}_{D} } \right)^{2} - 4{\Delta }T_{max} \left[ D \right]} }}{2}$ was used to estimate ∆T_max_ and K_D_. Nonlinear regression and sigmoidal dose–response curves (GraphPad PRISM8) were used to calculate IC_50_ values.

## Supplementary Information

Supplementary Information
