# Supplementary material for: Fluorescent thermal shift-based method for detection of NF-κB binding to double-stranded DNA
Source: Sci Rep. 2021 Jan 27;11:2331. doi: 10.1038/s41598-021-81743-1 (PMC7840993; doi:10.1038/s41598-021-81743-1)

Supplementary information

Fluorescent thermal shift-based method for detection of NF-κB binding to double-stranded DNA

Peter D. Leitner[1,2,3], Ilja Vietor[1], Lukas A. Huber[1,2], Taras Valovka[*1,4]

[1] Institute of Cell Biology, Biocenter, Medical University of Innsbruck, Innsbruck, Austria.

[2] Austrian Drug Screening Institute, ADSI, Innsbruck, Austria.

[3] Department of Biotechnology and Food Engineering, MCI Technik, Innsbruck, Austria.

[4] Department of Pediatrics I, Medical University of Innsbruck, Innsbruck, Austria.

Correspondence:

Taras Valovka

taras.valovka@i-med.ac.at

Department of Pediatrics I,

Medical University of Innsbruck,

Anichstrasse 35, 6020 Innsbruck, Austria.

Institute of Cell Biology, Biocenter,

Medical University of Innsbruck,

Innrain 80-82, 6020 Innsbruck, Austria.

Supplementary Figure 1. Analysis of the $p50_{1\text{-}367}$ protein using F-TSA based DNA binding assay (**a**) SDS-PAGE of recombinant HIS-tagged $p50_{1\text{-}367}$ and $p65_{1\text{-}306}$ proteins. (**b**) The thermal stabilization of $p50_{1\text{-}367}$ probe in the presence of 10 µM 1κB and 2κB dsDNA oligomers. Midpoint temperatures of the protein-unfolding transition (T_m) are presented as bars. Values are mean ± SD of three independent measurements (*** $p < 0.001$). (**c**) Thermal stability shift assessed with varying 2κB dsDNA concentrations. ΔTm, change in Tm of $p50_{1\text{-}367}$ probe caused by dsDNA. The curve is based on two separate measurements (open and closed circles).

Supplementary Figure 2. Stability and dsDNA binding of $p65_{1\text{-}306}$ at 4°C and 30°C. 10 µM of 2κB dsDNA were incubated with 1.5 µM $p65_{1\text{-}306}$ probe at 4°C or 30°C for 45 min prior to the thermal denaturation. Midpoint temperatures of the protein-unfolding transition (T_m) are presented as bars. Values are mean ± SD of three measurements (** $p < 0.01$ and *** $p < 0.001$).

Supplementary Figure 3. Evaluation of dose-dependent inhibition of p65/RelA and p50 by withaferin A. (**a**) The thermal denaturation of $p50_{1\text{-}367}$ was evaluated in the absence and presence of 10 µM 2κB dsDNA and different amounts of WFA, as indicated. Midpoint temperatures of the protein-unfolding transition (T_m) are presented as bars. Values are mean ± SD of three measurements (** $p < 0.01$). (**b**) IC_{50} curves for the WFA-mediated inhibition of $p65_{1\text{-}306}$ (left) and $p50_{1\text{-}367}$ (right). The curves are based on at least two separate measurements.

Supplementary Figure 4. *p*-XSC but not DGTS inhibits binding of p65 to dsDNA. (**a,b**) Chemical structures of *p*-XSC and DGTS are shown. The thermal stabilization of $p65_{1\text{-}306}$ by 2κB dsDNA (10µM) has been assessed upon the treatment with different concentrations of *p*-XSC and DGTS. Midpoint temperatures of the protein-unfolding transition (T_m) are presented as bars. Values are mean ± SD (* $p < 0.05$ and ** $p < 0.01$).

Supplementary Figure 5. Uncropped images. Dashed line designates the cropped area.

Supplementary Figure 1

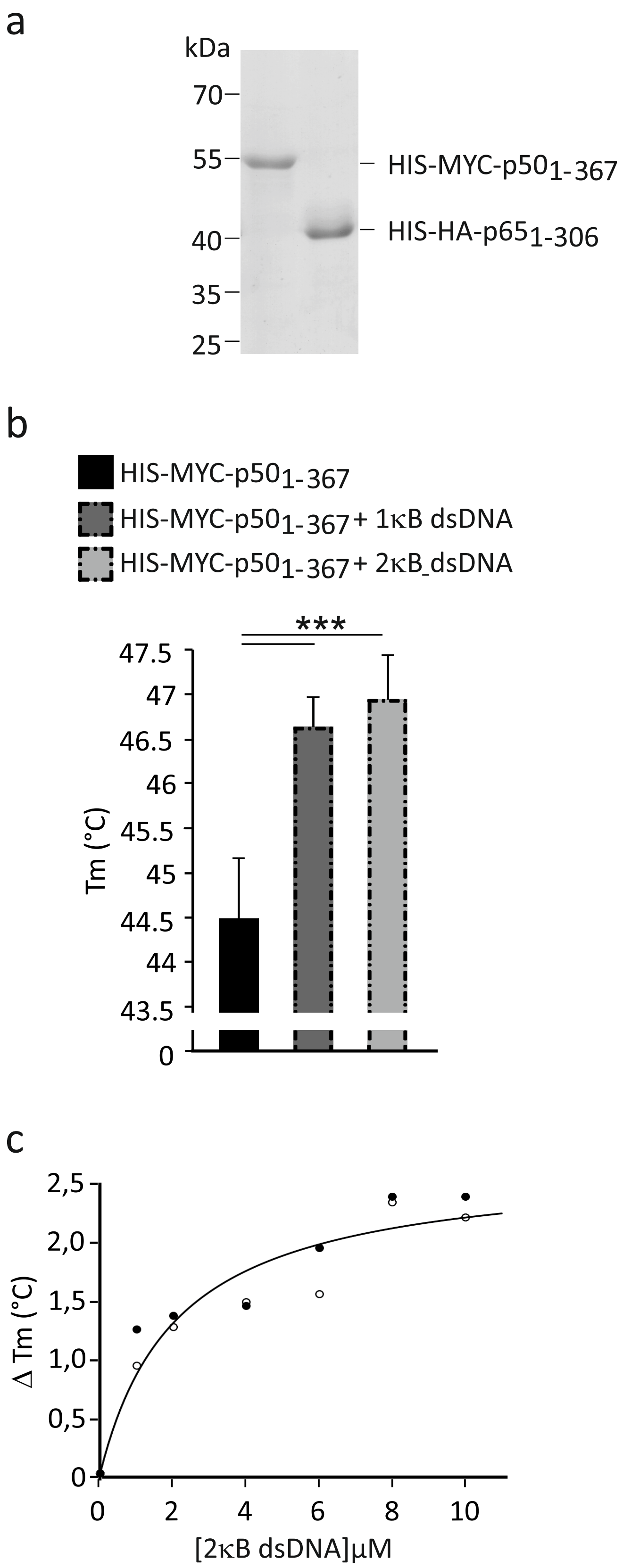

Supplementary Figure 2

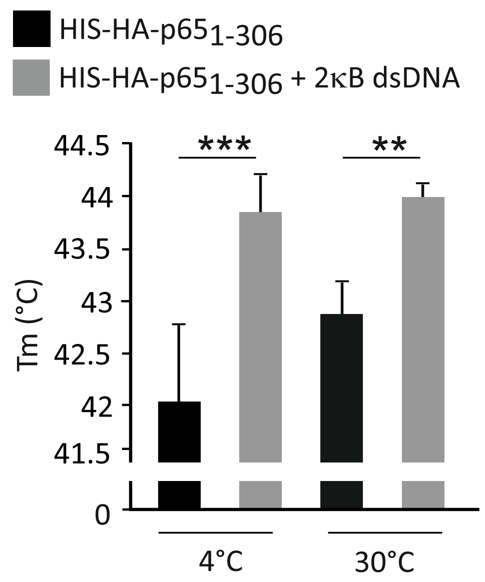

Supplementary Figure 3

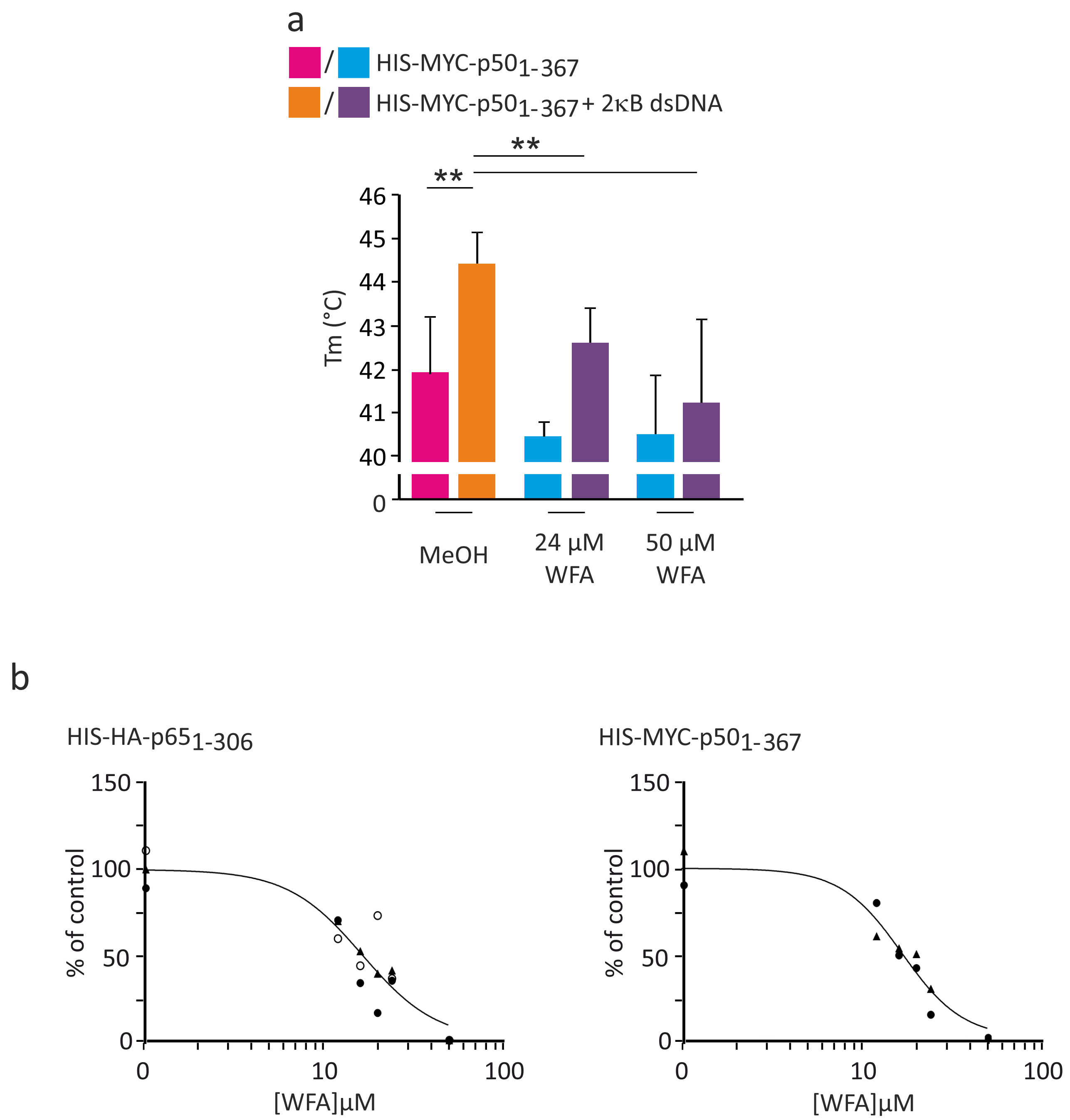

Supplementary Figure 4

a

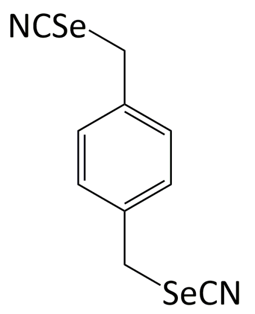

p-Xyleneselenocyanate (*p*-XSC)

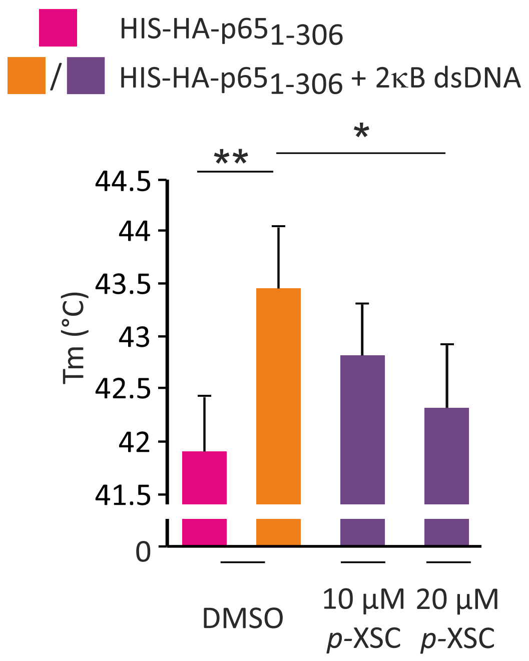

b

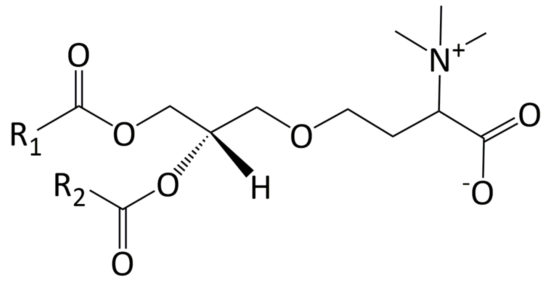

Diacylglyceryl-N,N,N-trimethylhomoserine (DGTS)

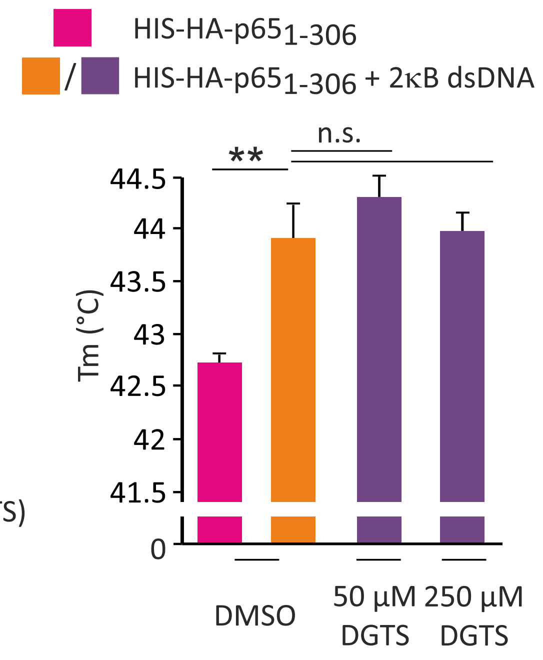

Supplementary Figure 5

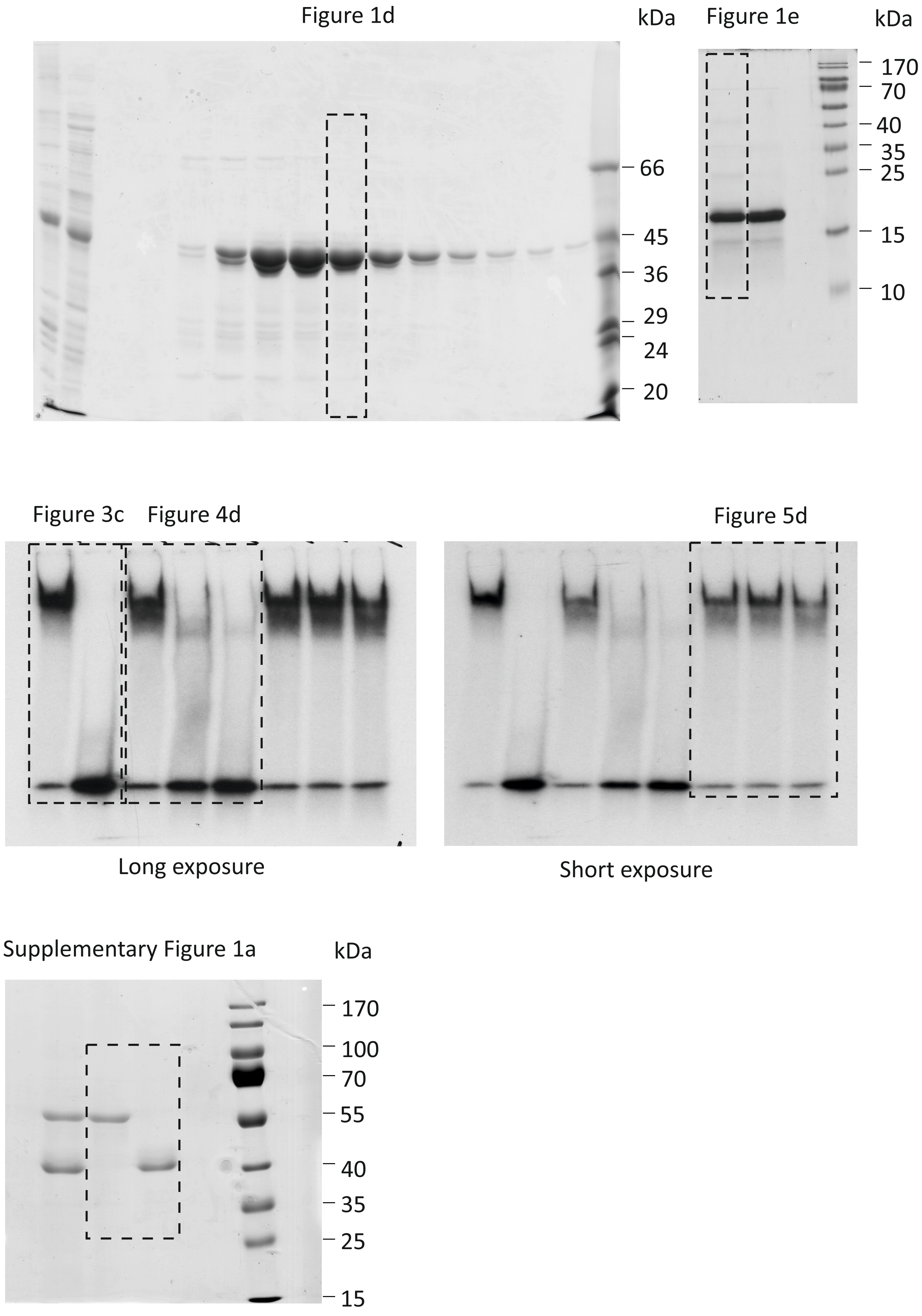

Supplement: Supplementary file 1 — Supplementary Information [file 41598_2021_81743_MOESM1_ESM.pdf]
